# Evaluation of the interaction between polymyxin B and *Pseudomonas aeruginosa* biofilm and planktonic cells: reactive oxygen species induction and zeta potential

**DOI:** 10.1186/s12866-019-1485-8

**Published:** 2019-05-29

**Authors:** Marlucy Rodrigues Lima, Gabriella Freitas Ferreira, Wallace Ribeiro Nunes Neto, Joveliane de Melo Monteiro, Áquila Rodrigues Costa Santos, Priscila Batista Tavares, Ângelo Márcio Leite Denadai, Maria Rosa Quaresma Bomfim, Vera Lúcia dos Santos, Sirlei Garcia Marques, Andrea de Souza Monteiro

**Affiliations:** 1 Faculdade de Ciências da Saúde, Universidade Vale do Rio Doce, Governador Valadares, MG Brazil; 2 Departamento de Farmácia, Programa Multicêntrico de Pós-Graduação em Bioquímica e Biologia Molecular, Universidade Federal de Juiz de Fora, UFJF, Campus Governador Valadares - MG. R. Manoel Byrro, 241 - Vila Bretas, Governador Valadares, MG 35032-620 Brazil; 3 Universidade CEUMA, São Luís, MA Brazil; 4 Departamento de Microbiologia, Instituto de Ciência Biológicas, Universidade Federal de Minas Gerais, Belo Horizonte, MG Brazil; 5 Hospital Universitário da Universidade Federal do Maranhão, São Luís, MA Brazil; 6 Laboratório Cedro, São Luís, MA Brazil

**Keywords:** *P. aeruginosa*, Biofilm and planktonic cells, Polymyxin B, Reactive oxygen species, Surface electrical property

## Abstract

**Background:**

Although the most widely accepted mechanism of action for polymyxins is related to bacterial lysis via disruption, we hypothesized that this antimicrobial drug class could have other effects on *Pseudomonas aeruginosa* planktonic and sessile cells. Little is known regarding oxidative burst and zeta potential (ZP) data associated with the interaction between polymyxin B and *P. aeruginosa* cells. The present study evaluated endogenous reactive oxygen species (ROS) production and changes in the net charges of biofilm and planktonic cells in response to polymyxin B.

**Results:**

Polymyxin B induced concentration-dependent killing at all concentrations tested in planktonic and sessile cells from *P. aeruginosa* strains. Sublethal concentrations of polymyxin B induced oxidative burst. ROS production was higher in resistant planktonic cells than in biofilm cells but this was not observed for susceptible cells. Moreover, no net surface charge alterations were observed in planktonic cells from a susceptible strain treated with polymyxin B, but a significant increase of ZP was noted in planktonic cells from a resistant strain.

**Conclusion:**

Oxidative burst generated by planktonic and sessile cells from *P. aeruginosa* strains against polymyxin B indicates that ROS may have an important role in the mechanism of action of this drug. ZP data revealed that electrostatic interactions of the cationic peptide with the anionic surface of the cells are strain-dependent. Therefore, we suggested that the intracellular effects of polymyxin B should be further investigated to understand polymyxin B-induced stress in *P. aeruginosa.*

## Background

*Pseudomonas aeruginosa* is one of the most important pathogenic bacteria species in the context of hospital infections [1]*.* It causes high mortality rates in immunocompromised patients, and it is currently classified as a “superbug” because of the limited effectiveness of antimicrobial drugs [2].

The emergence of *P. aeruginosa* with resistance to β-lactam antibiotics, including carbapenems, has resulted in few effective treatments for patients with serious infections [3–6]. Over the past 20 years, polymyxins have been reintroduced for the treatment of infections caused by gram-negative bacteria such as *P. aeruginosa* with multi-drug-resistant phenotypes, including resistance to carbapenems and quinolones [7].

Polymyxin B is a cationic antimicrobial cationic peptide that increases cell membrane permeability. The most widely accepted mechanism of action for polymyxins is the interaction with lipopolysaccharide (LPS) in the outer membrane of gram-negative bacteria, disrupting cellular osmotic balance [7] Thus, we hypothesized that polymyxins could have other effects on planktonic and sessile gram-negative bacteria. Little is known regarding the physicochemical effects of polymyxin B on *P. aeruginosa* biofilms, including the zeta potential (ZP). Electrostatic interactions have been observed between cationic agents and *Klebsiella pneumoniae*, *Acinetobacter baumannii*, *Staphylococcus aureus*, and *Escherichia coli* [8–10], and it was suggested that these interactions interfere with cellular net charges and consequently alter cell surface permeability, leading to cell death.

There is also a gap in knowledge regarding polymyxin B-induced endogenous reactive oxygen species (ROS) production in planktonic and sessile *P. aeruginosa* cells. For ciprofloxacin, the formation of hydroxyl radicals (^•^OH) in planktonic cells and biofilm-grown *P. aeruginosa* contributes to the mechanism of bactericide [11]. Sampson and colleagues observed that the rapid production of hydroxyl radicals caused by polymyxins is involved in *A. baumannii*, *E. coli*, and *Francisella novicida* planktonic cell death [12]. Conversely, some studies demonstrated that the bactericidal activity of colistin on planktonic and biofilm *P. aeruginosa* cells is independent of ^•^OH formation [13, 14]. Although these studies have produced conflicting results, various cell pathways have been proposed to be responsible for the generation of ROS triggered by the exposure of *E. coli* to antibiotics [15]. ROS increased the frequency of mutation rates in *gyrA* and *gyrB* in bacterial cells exposed to ampicillin, contributing to a norfloxacin-resistant phenotype [15]. ROS generation in *E. coli* associated with antibiotic exposure may also be caused by changes in redox complexes that contribute to cell damage and death because of the alterations to central metabolism, cellular respiration, and iron metabolism [16].

Thus, we evaluated ROS production in biofilm-derived and planktonic *P. aeruginosa* cells in response to polymyxin B and investigated differences in bacterial surface charges caused by polymyxin B as measured by zeta potential.

## Results

### Minimal inhibitory concentration (MIC), minimum biofilm eliminating concentration (MBEC), and time-kill curve assays for planktonic and biofilm cells

The MIC and MBEC for polymyxin B against *P. aeruginosa* P1C were 0.5 and 128 mg/L, respectively. For the P9C strain, these values were 64 and 512 mg/L, respectively. Because of the MIC, the P1C strain was classified as susceptible to polymyxin B, whereas the P9C strain was considered resistant [17].

**Fig. 1:**
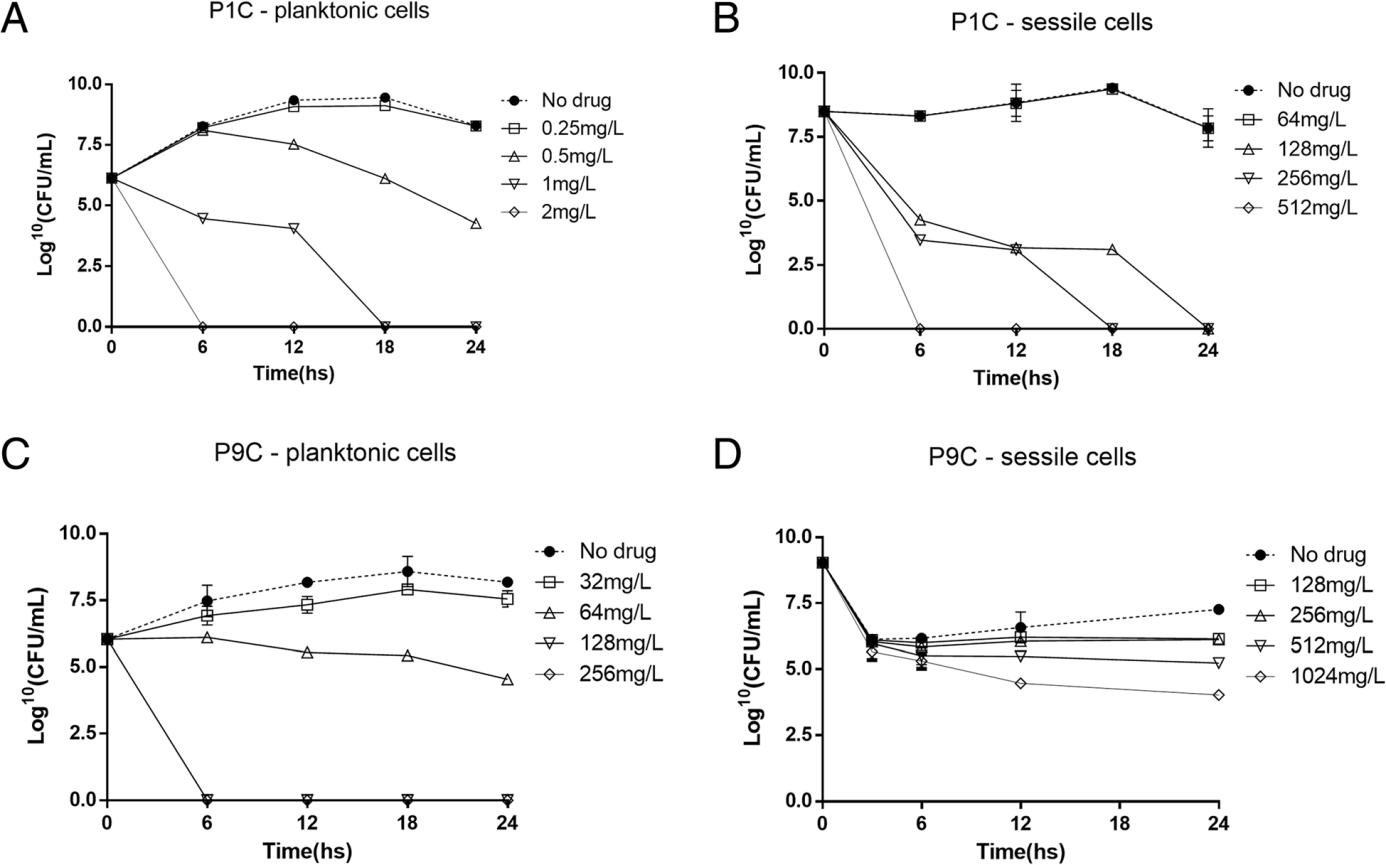
Polymyxin B time-kill curves against *Pseudomonas aeruginosa* strains. Time-kill curve generated using planktonic and biofilm cells from P1C (**a** and **b**) and P9C (**c** and **d**) at different polymyxin B concentrations. Results are expressed as log_10_ (CFU/mL)

To evaluate the kinetics of the action of polymyxin B against planktonic and sessile cells, time-kill curves were generated at different times and concentrations using two *P. aeruginosa* strains. For planktonic cells from the susceptible strain, polymyxin B provided a bacteriostatic curve at 0.5 mg/L (MIC), with 50% growth inhibition after 24 h of treatment (Fig. 1a). At concentrations of 1 mg/L (2× MIC) and 2 mg/L (4× MIC), polymyxin B induced a concentration-dependent killing of susceptible *P. aeruginosa* planktonic cells, with 100% growth inhibition after 18 h of treatment at 2× MIC and after 6 h of treatment at 4× MIC (Fig. 1a). Same profile was observed for resistant strain (Fig. 1c): bacteriostatic curve at 64 mg/L (MIC) and bactericidal curve at 128 (2× MIC) and 256 mg/L (4 × MIC). Conversely, polymyxin B induced concentration-dependent killing at all concentrations tested in sessile cells from both strains (Fig. 1b and d).

### ROS production

**Fig. 2:**
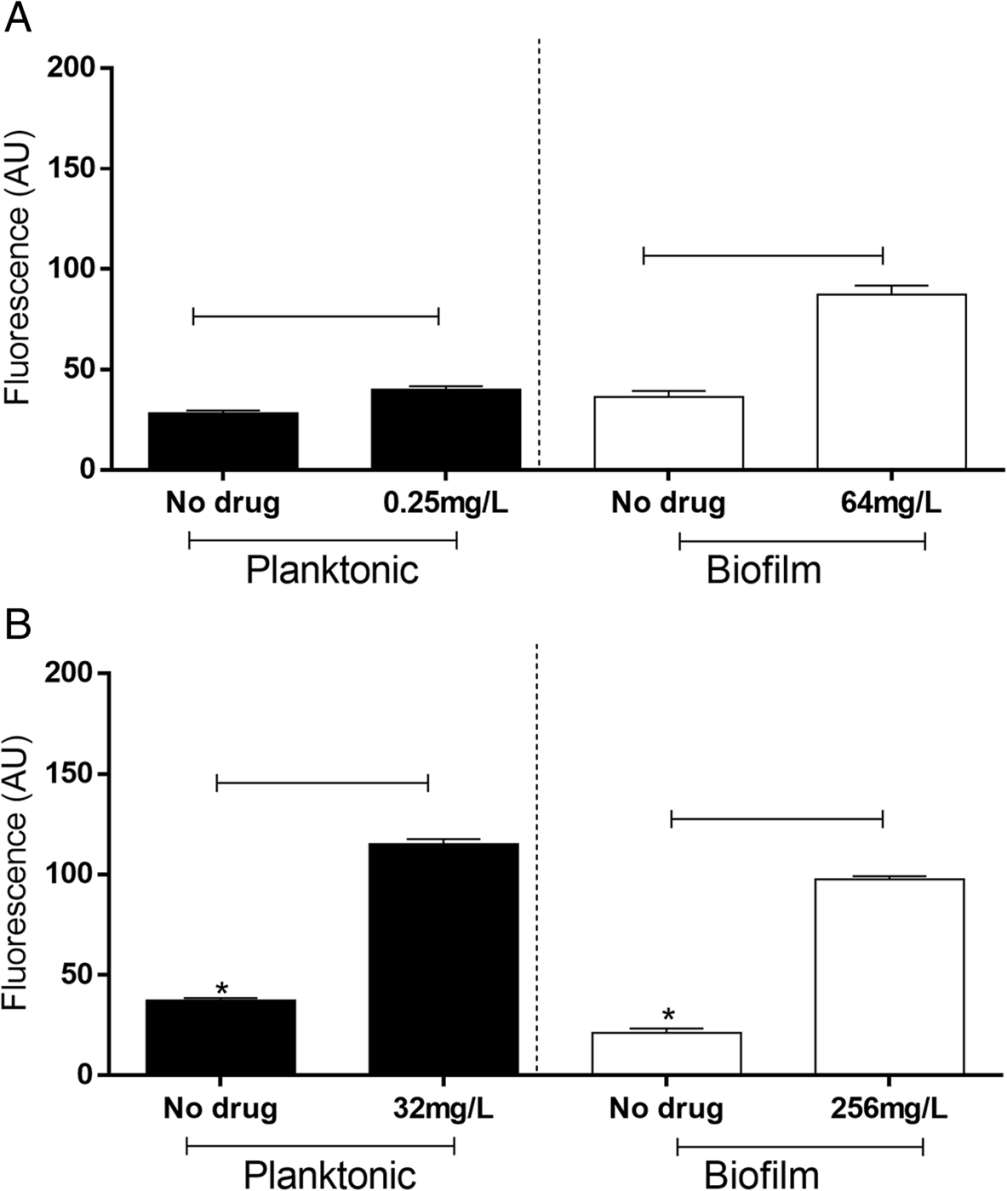
Quantities of reactive oxygen species (ROS) in *Pseudomonas aeruginosa* P1C (**a**) and P9C (**b**) cells in the presence of polymyxin B. ROS were quantified at 37 °C after 3 h of incubation. The groups contained untreated and treated cells with 0.5× minimum inhibitory concentration of polymyxin B and 1% hydrogen peroxide (data no shown). Results are expressed in arbitrary units of fluorescence. Each data point represents the mean of three independent samples. Significant differences between the treated and untreated cells are represented by connected lines (*P* < 0.05), and those between planktonic and biofilm cells are represented by asterisks (*P* < 0.05). Data are presented as the mean ± S.E. of two independent experiments

Polymyxin B significantly induced ROS production after 3 h of treatment in planktonic (P1C: untreated, 28.20 ± 1.44 AU; treated, 40.07 ± 1.61 AU; P9C strain: untreated, 37.38 ± 1.23 AU; treated, 115.00 ± 2.5 6 AU) and sessile cells compared with the findings for the growth control (P1C: untreated, 36.36 ± 3.13 AU; treated, 87.24 ± 4.47 AU; P9C strain: untreated, 21.24 ± 2.13 AU; treated, 97.61 ± 1.50 AU) (P < 0.05) (Fig. 2a and b).

The results illustrated that ROS production was higher in resistant planktonic cells than in biofilm cells (P < 0.05) (Fig. 2b), but this was not observed for susceptible cells (Fig. 2a).

### Effect of polymyxin B on *P. aeruginosa* cell surface zeta potential (ZP)

**Fig. 3:**
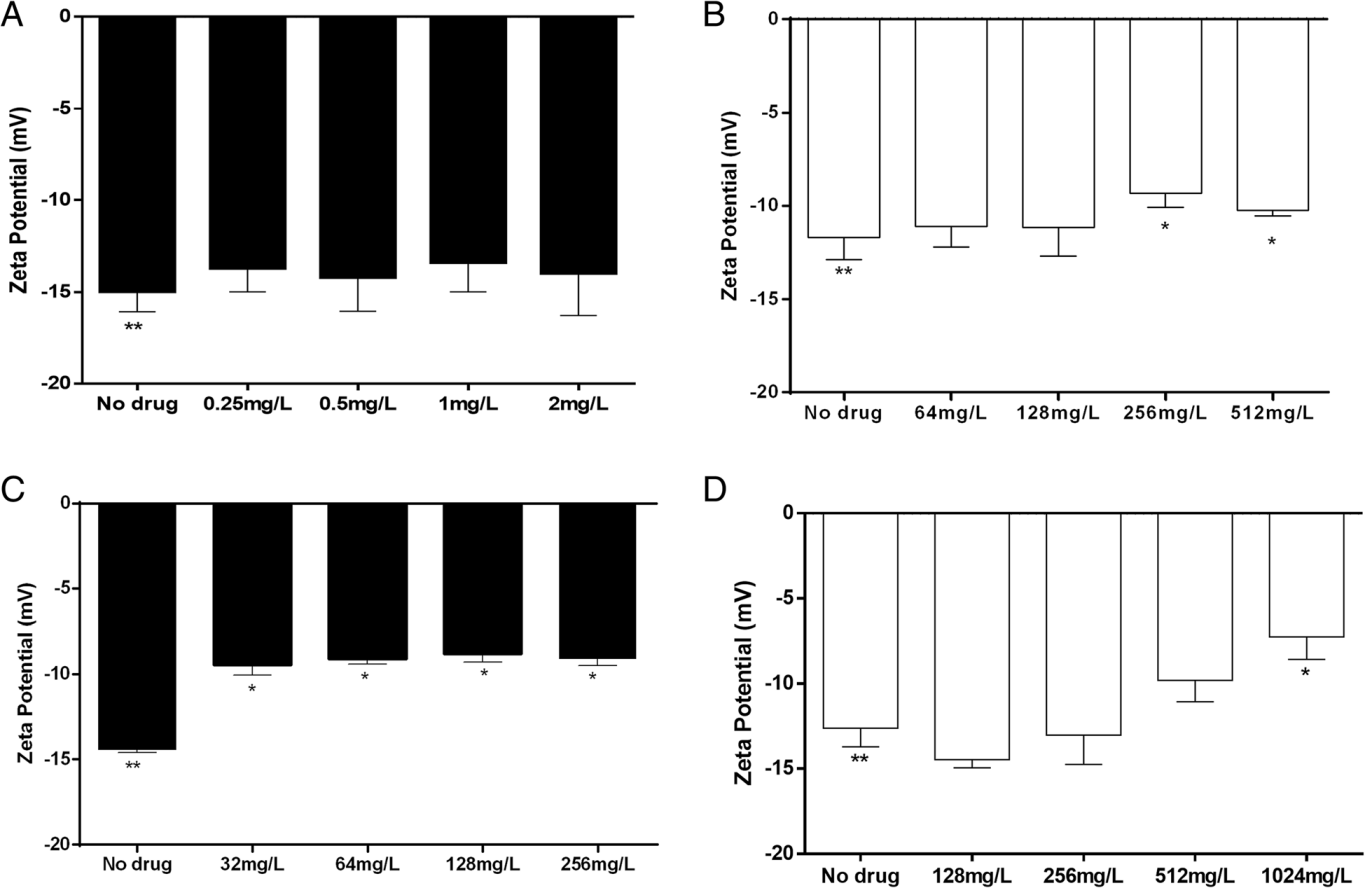
Determination of the interaction between polymyxin B and *Pseudomonas aeruginosa* P1C planktonic cells (**a**), P1C biofilm cells (**b**), P9C planktonic cells (**c**), and P9C biofilm cells (**d**) using the zeta potential (ZP/mV). Significant differences between the treated and untreated cells are represented by one asterisk (*P* < 0.05). Significant differences between no treatment planktonic and biofilm cells are represented by two asterisks (*P* < 0.05). Data are presented as the mean ± S.E. of two independent experiments

For planktonic cells from susceptible strains, 0.25 (0.5× MIC), 0.5 (MIC), 1 (2× MIC) and 2 mg/L (4× MIC) polymyxin B did not change the electrical charges of surface cells (untreated, − 14.96 ± 1.11 z/mV; 0.25 mg/L, − 13.68 ± 0.53 z/mV; 0.5 mg/L, − 14.18 ± 0.76 z/mV; 1 mg/L, − 13.38 ± 0.65 z/mV; 2 mg/L: − 13.95 ± 0.94 z/mV) (Fig. 3a). Conversely, for sessile cells, 256 (2× MBEC) and 512 mg/L (4× MBEC) polymyxin B modified the net charges of their surfaces (untreated, − 11.70 ± 0.48 z/mV; 128 mg/L, − 11.11 ± 0.45 z/mV; 256 mg/L, − 11.17 ± 0.67 z/mV; 512 mg/L, − 9.33 ± 0.30 z/mV; 1024 mg/L, − 10.24 ± 0.30 z/mV) (Fig. 3b).

We observed that the electrical charges of the surfaces of resistant planktonic cells were modified significantly following exposure to all polymyxin B concentrations tested (untreated, − 14.45 ± 0.17 z/mV; 32 mg/L, − 9.45 ± 0.61 z/mV; 64 mg/L, − 9.08 ± 0.32 z/mV; 128 mg/L, − 8.79 ± 0.50 z/mV; 256 mg/L, − 9.12 ± 0.37 z/mV) (P < 0.05) (Fig. 3c). However, alterations of ZP were observed in cells detached from the biofilm and treated with polymyxin B only at a concentration of 1024 mg/L (4× MIC) (untreated, − 12.63 ± 0.48 z/mV; 128 mg/L, − 14.48 ± 0.47 z/mV; 256 mg/L, − 13.03 ± 0.77 z/mV; 512 mg/L, − 9.81 ± 0.56 z/mV; 1024 mg/L, − 7.25 ± 0.59 z/mV) (Fig. 3d).

## Discussion

Antimicrobial resistance in *P. aeruginosa*, as indicated by biofilm formation, is recognized as a major cause of therapeutic failure for chronic lung infection in patients with cystic fibrosis [18, 19]. Polymyxin B is increasingly used clinically as the last therapeutic option for multidrug-resistant gram-negative bacterial infections [20, 21]. Consequently, more studies are been conducted to understand the effect of polymyxins on *P. aeruginosa*.

As expected, biofilm cells were more resistant to polymyxin B than planktonic cells. Usually, mature biofilm expresses an extracellular matrix that functions as a protective barrier against antibiotic permeation [22]. This is partially caused by metabolic changes resulting from the limited oxygen availability and nutrient penetration through the biofilm because of bacterial consumption.

Only a small number of in vitro time-kill studies on polymyxins against *P. aeruginosa* strains have been conducted. To the best of our knowledge, this is the first study examining the pharmacodynamics of polymyxin B in sessile cells from *P. aeruginosa*. Our results demonstrated that the bactericidal activity of polymyxin B was concentration-dependent, corroborating other studies [20, 23]. Significant declines (> 2-log decrease) in the bacterial burden were observed after 24 h after MIC and MBEC for both strains. With increasing polymyxin B concentrations, a higher killing rate and a greater extent of killing were observed in both strains for planktonic and sessile cells (Fig. 1).

Studies have illustrated that some antibiotics promote oxidative burst in microorganisms, but their main mechanisms of action are not related with reactive species [15, 24–26]. The role of oxidative burst in the effects of polymyxin against *P. aeruginosa* is not clear. Some studies reported that the bactericidal activity of colistin in *P. aeruginosa* is independent of hydroxyl radical formation [14], and the effect was increased in biofilms under anaerobic conditions [13]. However, other studies demonstrated that bacterial respiration can be inhibited by polymyxins because of cytoplasmic membrane disruption in *P. aeruginosa* cells, allowing the accumulation of toxic intermediates [27–29]. Our results corroborate the hypothesis that treatment of *P. aeruginosa* cells with polymyxin B results in increased ROS levels (Fig. 2). It has been proposed that polymyxin initially accumulates in the outer membrane and subsequently penetrates the inner membrane to enter the cytoplasm [30], leading to reactive species production [31, 32] via inhibition of NADH-quinone oxidoreductase activity [29]. The free radicals accumulate and damage DNA, lipids, and proteins, eventually causing cell death [16].

The production of ROS was higher in planktonic cells than biofilm cells for the resistant strain (Fig. 2b) regardless of treatment with polymyxin B. However, this was not true for the susceptible strain (Fig. 2a). The intrinsic oxidative burst and activity of antioxidant cell system may be strain-dependent [33]. Based on our results, we can hypothesize that sessile cells from the P9C strain is better adapted to oxidative stress than planktonic cells, which may explain the lower damage caused by polymyxin B-induced stress.

It is unclear in the literature whether polymyxin B can change the net charges of planktonic and sessile surface cells. It has been suggested that alteration of ZP may be correlated with the enhancement of membrane permeability [9]. We observed that electrical charges of the surfaces of resistant planktonic cells were modified following exposure to all concentrations of polymyxin B tested (Fig. 3c), but no alterations of ZP were found in planktonic cells from the susceptible strain (Fig. 3a). The net surface charge must change during ionic interactions, as shown by the ZP measurements. We believed that cell death caused by polymyxin B in the *P. aeruginosa* P1C susceptible strain is principally mediated by anionic intracellular target(s) of the polymyxins, probably through oxidative burst, but more studies needed to confirm this hypothesis. Prior research demonstrated that polymyxin-mediated cell death can occur prior to cytoplasmic membrane depolarization, and the interaction with membranes may not the only lethal event [30, 34]. Consequently, we believe that for resistant *P. aeruginosa* P9C, the interaction of polymyxin B with LPS in the outer membrane limited the penetration of this drug into the cytoplasm, diminishing the antimicrobial effect of polymyxin B. Corroborating this hypothesis, a prior study reported that a polymyxin-resistant strain selected from the reference *K. pneumoniae* strain in the presence of colistin had a less negative and more permeable outer membrane than the wild-type strain [8]. In this case, the increased membrane fluidity did not coincide with polymyxin susceptibility [8, 34, 35].

Cells detached from biofilms (Fig. 3b and d) from both strains had less negative charges than their respective planktonic cells (Figs. 3a and c). Previous studies demonstrated that a strategy used by bacteria to adapt to polymyxin B-mediated stress involves conversion of the surface charge to a more positive value through LPS modifications. These changes reduce or prevent the initial interaction between the antibiotic and LPS, resulting in resistance [26]. Some bacterial species have evolved different mechanisms to modify lipid A, thereby reducing the net negative cell surface charge. The principal modification consists of the insertion of 4-amino-4-deoxy-l-arabinose and/or phosphoethanolamine in the phosphate groups of the molecule [36–38]. It was observed that the net cell charge is influenced by the cell growth phase [8], and thus, we supposed that phenotypic modifications of planktonic and sessile cells are responsible for our data. Clearly, the relationship between polymyxin resistance and ZP is extremely complex.

We observed that a higher concentration of polymyxin B is required to overcome the extracellular matrix of the biofilm and promote changes of the net charges of cells (Fig. 3b and d). In addition, once the extracellular matrix is formed, it can form electrostatic interactions (repulsion and sequestration molecules) with polymyxin B, decreasing the amount of free drug interacting with the surface of gram-negative bacteria [39, 40].

## Conclusions

Oxidative burst generated by *P. aeruginosa* cells in response to polymyxin B may have an important role in mechanism of action of this drug. The zeta potential data revealed that the electrostatic interactions of polymyxin B with the cell surface are strain-dependent, and they could be related to the mechanism of cell killing, which is probably attributable to extracellular and intracellular processes.

## Methods

### Antimicrobial drug susceptibility testing and time-kill curves for planktonic cells

In this study, tests were performed using the clinical *P. aeruginosa* strains P1C and P9C obtained from the culture collection of the Applied Microbiology Laboratory of the University CEUMA (Maranhão, São Luís, MA, Brazil). *P. aeruginosa* P1C and P9C strains were isolated from blood culture samples and identified via MALDI-QTOF-mass spectrometry (Biotyper system, Bruker, Billerica, MA, USA) in the Cedro Laboratory (Maranhão, São Luís, MA, Brazil)*.* In addition, P1C was susceptible to polymyxin B, cefepime, and ceftazidime; indifferent to ciprofloxacin; and resistant to levofloxacin. *P. aeruginosa* The P9C strain was resistant to norfloxacin, ciprofloxacin, meropenem, ampicillin, and polymyxin B and was chosen for the study among 40 total tested isolates because of its excellent biofilm formation pattern in vitro (data not shown). The P9C strain was maintained on slants of brain heart infusion agar (BHIA; Difco Laboratories, Detroit, MI, USA) at 4 °C. Prior to each test, the strain was subcultured on BHIA for 24 h at 35 °C.

The MIC of polymyxin B in planktonic cells was determined using the broth microdilution method as described in CLSI M07-A10 [17]. For this purpose, different concentrations of polymyxin B (0.125–512 mg/L) were incubated with 100 μL of cation-adjusted Mueller-Hinton broth (Difco) supplemented to give final concentrations of calcium 20–25 mg/mL and magnesium 10–12.5 mg/L in 96-well plates. The bacterial inoculum was prepared in 0.9% NaCl (sterile saline), and transmittance of the suspensions was adjusted to 75–77% (530 nm), followed by further dilution in cation-adjusted Mueller-Hinton broth to achieve a concentration of 5.0 × 10^5^ CFU/mL in the well. Negative (no bacteria) and growth (no drug) controls were included for each assay. The plates were incubated under aerobic conditions at 35 °C for 24 h. The MIC endpoint for interpreting the results was determined visually as 100% reduction of growth in the presence of polymyxin B compared with the control growth. The results were confirmed by adding the salt 3-(4,5-dimethylthiazol-2-yl)-2,5-diphenyltetrazolium bromide (1.0 g/L, MTT, Sigma-Aldrich) to determine the reduction of the cell metabolic activity as described previously. All the tests were performed in triplicate.

The time-kill curve assays for planktonic cells were performed as described previously [41] with modifications. Bacterial inoculum prepared to achieve a final concentration of 5.0 × 10^5^ CFU/mL were treated with polymyxin B were prepared cation-adjusted Mueller-Hinton broth and added to the wells.

A 50-μL aliquot of bacterial inoculum was removed from the microtiter plates containing polymyxin B at different intervals until 24 h and serially diluted in sterile saline (NaCl, 0.9%), before plating on *Pseudomonas* agar plates (*Difco*, Kansas City, *MO, USA*) for colony count determinations. The plates were incubated at 35 °C for 24 h prior to colony counting. The data are presented as the means of three independent experiments in duplicate assays.

#### MBEC assay and time-kill curve assay for biofilm cells

Biofilms were formed overnight at 37 °C in non-treated 96-well polystyrene plates. Bacterial concentrations were prepared as described previously [42]. Next, biofilms were washed three times and exposed to 200 μL of polymyxin B was diluted in cation-adjusted Mueller-Hinton broth to concentrations of 0.125–512 mg/L. Plates were incubated overnight at 37 °C. MTT salt (1.0 g/L) was used to assess anti-biofilm activity after overnight incubation at 37 °C (as described previously) [47]. The lowest concentration at which MTT was not converted to formazan was considered the MBEC. This experiment was performed in triplicate.

For the time-kill curve assay for biofilm cells, biofilms were washed three times and exposed to polymyxin B diluted in cation-adjusted Mueller-Hinton broth. The plates were incubated at 37 °C until 24 h and serially diluted in sterile saline (NaCl, 0.9%), before plating on *Pseudomonas* agar plates (*Difco*, Kansas City, *MO, USA*) for colony count determinations. The plates were incubated at 35 °C for 24 h prior to colony counting. The data are presented as the means of three independent experiments in duplicate assays.

### Measurement of ROS production

Endogenous ROS were measured using a fluorometric assay as described previously with modifications [25, 43]. Planktonic or biofilm cells (1.0 × 10^7^ CFU/mL) were treated with polymyxin B (planktonic, 0.5× MIC; biofilm, 0.5× MBEC) or hydrogen peroxide (1% positive control) in a buffered mineral medium (in g/L: 1.5 K_2_HPO_4_, 0.5 KH_2_PO_4_, 0.5 NaCl, 0.5 MgSO_4_·7H_2_O, 3.0 NH_4_NO_3_, 0.002 FeSO_4_·7H_2_O, 0.002 CaCl_2_·2H_2_O, 0.2 yeast extract, and 5% glucose; pH 7) and incubated with 10 μM 2′,7′-dichlorofluorescin diacetate (Invitrogen, Life Technologies, Carlsbad, CA, USA) at 37 °C. After 3 h of incubation, fluorescence was measured using a Varioskan™ LUX multimode microplate reader (Thermo Fisher Scientific Inc., Waltham, MA, USA) using excitation and emission wavelengths of 485 and 535 nm, respectively. Data were expressed as arbitrary units of fluorescence ± standard error of two independent experiments. Cells incubated in the absence of polymyxin B were used as controls.

Statistical analyses were performed using GraphPad Prism ver. 5.00 for Windows (GraphPad Software, San Diego, CA, USA). The Kruskal–Wallis statistical analysis was used to compare differences among groups, followed by Dunn’s multiple comparison post-test to detect specific differences. *P* < 0.05 was considered significant.

### Effect of polymyxin B on zeta potential (ZP)

Planktonic and biofilm cells were used in the assays. The method used was described previously with modifications [44]. After careful washing of the biofilms with sterile 0.9% NaCl, the cells were detached, suspended in 5 mL of 0.9% NaCl, and vortexed until visual observation of the complete dissolution of the lumps. These suspensions were centrifuged (4000×*g* for 10 min), and the resulting cell pellet was resuspended in 2 mL of 0.9% NaCl for optical density determination using a spectrophotometer. To obtain planktonic cell suspensions, the bacteria were grown in brain heart infusion broth and washed with sterile 0.9% NaCl. The assay included titration of four manual injections with aqueous polymyxin B in a glass beaker containing *P. aeruginosa* cell suspensions (planktonic and biofilm cells) at an optical density at 600 nm (OD_600_) of 0.1. After each titration, the solution was transferred to a cuvette (Cell-Folded Capillary DTS1060; Malvern Instruments), and the ZP was determined. Data were expressed as mV ± standard error of two independent experiments.

The assays for determining ZP were conducted at 25 °C using a Zetasizer NanoZS instrument (Malvern Instruments Inc., Westborough, MA, USA) with 64 channels and a correlator at 633 nm. The technique used for the ZP measurements was in accordance with the Malvern laser Doppler velocimetry patterns coupled with M3-phase analysis light scattering.

Statistical analyses were performed using GraphPad Prism ver. 5.00 for Windows. One-way analysis of variance was used to compare differences among groups, followed by Bonferroni’s post-test to detect specific differences. *P* < 0.05 was considered significant.

## Abbreviations

AU: Arbitrary units
CFU: Colony formation unity
CLSI: Clinical and Laboratory Standards Institute
DNA: Deoxyribonucleic acid
LPS: Lipopolysaccharide
MALDI-QTOF: Matrix-assisted laser desorption/ionization
MBEC: Minimum biofilm eliminating concentration
MIC: Minimal inhibitory concentration
MTT: 3-(4,5-dimethylthiazol-2-yl)-2,5-diphenyltetrazolium bromid
NADH: Nicotinamide adenine dinucleotide
OD: Optical density
OH: Hydroxyl radicals
ROAS: reactive oxygen species
ZP: Zeta potential
